# Parental traits, reproductive investment, and genetic relatedness shape offspring development and morphology in an explosive-breeding toad

**DOI:** 10.1186/s12983-026-00627-0

**Published:** 2026-08-11

**Authors:** Chaoyang Wang, Yanlan Long, Xinlan Cheng, Xiang Fu, Panpan Liu, Qianwei Yang, Jingjing Yan, Jianghui Guo, Zhenhua Luo

**Affiliations:** 1 Institute of Evolution and Ecology, School of Life Sciences, Central China Normal University, Wuhan, Hubei 430079 China; 2 Liuzhou Chongyuan Middle School, Liuzhou, Guangxi 545616 China; 3 Xiamen No. 3 Middle School of Fujian, Xiamen, Fujian 361006 China; 4 Taiyuan No. 11 Middle School, Taiyuan, Shanxi 030008 China; 5 Middle School Attached to Luoyang Normal University, Henan Luoyang, 471023 China; 6 Changzhou Longcheng Senior High School, Changzhou, Jiangsu 213002 China

**Keywords:** Body size, *Bufo gargarizans*, Genetic relatedness, Growth rate, Parental effects

## Abstract

**Background:**

Parental effects link parental phenotype and environmental variation to offspring performance and evolutionary trajectories. However, their role remains poorly resolved in species lacking post-oviposition care, where offspring development is shaped mainly by pre-hatching parental influences. We investigated how parental phenotype, reproductive investment, and parental relatedness were associated with offspring development in the Asiatic toad (*Bufo gargarizans*). Amplectant pairs were collected from the wild, and offspring were reared under standardized laboratory conditions from fertilization to metamorphosis. Parental relatedness was estimated using microsatellite markers to evaluate how parental genetic similarity was associated with offspring developmental and morphological traits.

**Results:**

We detected positive assortative pairing by body mass in *B. gargarizans* and strong clutch effects across offspring developmental traits, indicating substantial among-family variation. After distinguishing among parental body size, body mass, and body condition, the most consistent associations involved paternal body condition, paternal investment, and parental relatedness. Paternal body condition was positively associated with offspring growth rate during the G25-G42 interval, suggesting faster mid-stage larval growth in offspring of males in better condition. Paternal reproductive investment was positively associated with post-metamorphic morphology, particularly tibiofibula length at G46. Parental relatedness was negatively associated with length-related morphology around metamorphosis, and this association varied among developmental stages. Maternal traits showed more limited and mainly stage-dependent associations with offspring traits.

**Conclusions:**

Our results show that offspring development in *B. gargarizans* is associated with parental phenotype, reproductive investment, and parental relatedness. Paternal body condition and paternal investment were associated with offspring growth and post-metamorphic morphology, whereas parental relatedness was negatively associated with length-related morphology around metamorphosis. These findings suggest that parental effects in this species are trait-specific and stage-dependent. They also highlight the importance of integrating paternal phenotype and parental relatedness into studies of amphibian reproductive ecology, especially in species without post-oviposition care.

**Supplementary Information:**

The online version contains supplementary material available at https://doi.org/10.1186/s12983-026-00627-0.

## Background

Parental effects are influences of parental traits, conditions, or their environments on offspring phenotype and fitness, which can be mediated through non-Mendelian pathways [1]. Such effects can transmit information across generations via differential nutrient allocation, hormonal provisioning, behavioral care, or environmental modification [2, 3]. In essence, parental effects constitute a form of transgenerational plasticity [4]. Theoretical and empirical works have shown that parental effects evolve under natural selection as adaptive mechanisms, especially when environments are at least partly predictable [5–7]. These effects allow parents to “encode” their perceived environmental states into gametes or the early conditions of offspring [8, 9]. In this way, they shape offspring phenotype before offspring directly experience that environment and can enhance subsequent survival and reproductive performance [3, 5, 10]. However, parental effects are not synonymous with simple genetic correlation or “maternal noise” [4]. On the one hand, phenotypic correlations among parents and offspring may arise either from environmentally mediated parental effects or from additive genetic effects, and these two sources must be carefully disentangled in both theoretical and experimental work [4, 11]. On the other hand, parental effects are not necessarily adaptive. If the correlation between the environment experienced by parents and that encountered by offspring is disrupted, or if parental condition deviates from the range favored by natural selection, the “environmental information” provided by parents may even reduce offspring fitness [2]. For example, in the Mexican spadefoot toad (*Spea multiplicata*), studies have shown that very high maternal body condition (approaching obesity) under captive conditions is associated with reduced tadpole survival. This pattern has been interpreted as a non-adaptive parental effect induced by obesity [12]. Against the backdrop of global change and intensive human alteration of habitats, a key challenge is to clarify in a systematic way how parental effects modulate offspring phenotypes and fitness across different environmental contexts. Addressing this challenge will improve our understanding of population persistence of animals and their evolutionary potential.

Research on parental effects in animals has long shown a bias toward mothers [1]. Some studies focused on how maternal body size and body condition influence early growth and survival of offspring by altering reproductive investment, such as clutch size, egg size, and egg quality [13, 14]. In several anuran and Caudata species, larger females or females in better body condition generally produce more and larger eggs, which in turn lead to larger hatchlings or higher offspring survival [15–17]. For example, in territorial eastern red-backed salamanders (*Plethodon cinereus*), female body length not only predicts clutch size and offspring body length but is also positively associated with long-term offspring survival under experimental conditions [16]. Similar patterns, where larger females produce more or larger offspring, have been reported repeatedly in some frogs and toads [15, 18]. These findings are regarded as important evidence for links among female choice, body-size evolution, and reproductive strategies [14, 19]. In contrast, the roles of paternal traits in reproductive success and offspring development have been underestimated, particularly in species without parental care, where males are often viewed simply as providers of sperm [20]. Evidence from some vertebrate taxa shows that body size, body condition, age, and nutritional or social environments of males can have “lasting effects” on offspring growth rate, physiological state, and even lifetime fitness [21]. These effects may act through changes in sperm quality, epigenetic marks on gametes, or seminal fluid composition [14, 20, 22]. In amphibians without post-oviposition parental care, recent experiments on the spiny toad (*Bufo spinosus*) have shown that paternal body size and body condition can explain substantial variation in several key offspring traits including embryonic developmental duration [23]. These results suggest that paternal effects in this group might be nonnegligible. Therefore, to obtain a comprehensive understanding of how parental effects shape offspring growth and development, maternal and paternal phenotypic traits need to be included together in a single analytical framework, and their relative contributions and stage-specific influences on offspring fitness must be compared in a systematic way.

Relatedness is an important factor influencing offspring growth and development, particularly when high parental relatedness results in inbreeding [24]. Inbreeding increases genomic homozygosity and reduces heterozygosity in offspring [25]. As a consequence, recessive deleterious mutations are more likely to be expressed, while heterozygote advantage may be weakened [26]. These genetic effects can affect multiple fitness-related traits, including birth mass, survival, and tolerance to pathogens and abiotic stressors [27]. Evidence from field studies and meta-analyses indicates that inbreeding depression is widespread in natural populations and is often expressed early in life through increased mortality or reduced growth [25]. The effects of inbreeding are strongly dependent on environmental conditions [28]. Under limited resources, increased competition, or other stressful environments, fitness losses caused by inbreeding are often amplified [29]. By contrast, under relatively benign laboratory conditions, these negative effects may be partially masked [30]. Crosses between geographically distinct populations can also result in outbreeding depression, whereby hybrid offspring show reduced performance [31]. For example, interpopulation crosses in the common frog (*Rana temporaria*) have produced tadpoles with smaller body size and higher rates of malformation [32]. Therefore, relatedness should be treated as an independent factor when evaluating parental effects on key offspring developmental traits, allowing clearer separation of parental effects, relatedness effects, and their interactions on offspring growth and development.

Frogs and toads that lack post-oviposition parental care and show communal breeding provide good ecological models for studying parental effects [33, 34]. Reviews have noted that communal egg-laying is widespread in reptiles and amphibians, and that, in most species, males and females do not provide direct care to eggs or offspring once oviposition and external fertilization are completed [35, 36]. Thus, parental influences on fertilization, hatching, and larval development might be expressed mainly through pre-oviposition investment in gamete quality and reproductive allocation in these taxa, rather than being confounded with complex parental care behaviors [37]. In such systems, eggs and tadpoles from different parental combinations can be reared under controlled experimental conditions. This makes it possible to assess how parental phenotypes and relatedness affect offspring growth rate, developmental duration, and morphological traits [34, 38].

Another important but still insufficiently addressed question is how maternal traits, paternal traits, and parental relatedness influence offspring growth and development across developmental stages. Most studies have focused on links between maternal body size or condition and fecundity, egg size, and offspring size. In contrast, paternal traits and parental relatedness are often examined separately, and their effects vary widely among species and environmental contexts. To address these gaps, the Asiatic toad (*Bufo gargarizans*) provides a suitable study system [39]. It breeds in aggregations in natural waterbodies and provides no parental care after oviposition. This reproductive biology allows offspring development to be monitored under standardized conditions [40, 41]. After natural mate choice in the field, amplexing pairs were captured and transferred to the laboratory. Under standardized conditions, offspring development was monitored from fertilization through metamorphosis. This design allowed us to test the effects of multiple parental traits on key developmental traits. Mixed-effects models were used to account for the hierarchical structure of the data. Because parental traits reflect both genetic and environmental influences, these components cannot be separated in our design. Therefore, our results should be interpreted as overall parental effects. This study provides a foundation for future work that combines genetic and environmental manipulations to clarify the mechanisms involved.

## Materials and methods

### Study species and sampling design

The Asiatic toad (Anura, Bufonidae) is one of the most widespread and abundant amphibian species in China. It is a typical explosive breeder and its breeding season generally spans January to April, with the reproductive activity peaking over only a few days. Fertilized eggs and tadpoles develop in water, and metamorphosis is typically completed within 2–3 months after fertilization [42]. Field sampling was conducted from February to March in 2019 at a permanent lentic pond (E110°3′39.96″, N29°46′18.25″) in the Badagongshan National Nature Reserve, China. This pond is a stable breeding site for the local wild population of the Asiatic toad. Throughout the breeding season, we surveyed the pond daily and recorded reproductive behaviors of the toads. When an amplexing pair was encountered, we captured, marked, and measured both individuals and then transferred the pair to a mesh enclosure placed in the pond to minimize disturbance to ambient water conditions and the social environment. After oviposition was completed, we collected ~ 10 cm sections of each egg string from the proximal, middle, and distal portions as experimental samples. The remaining egg strings and the parental individuals were returned to the pond the same day. Overall, we obtained egg strings from 20 pairs.

### Parental traits and genetic relatedness

All experiments were conducted under laboratory conditions. Snout-vent length (SVL) and body mass of amplexing males and females were measured using digital calipers (precision 0.01 mm) and an electronic balance (precision 0.01 g), respectively [43]. Measurements were taken before and after oviposition. Each trait was measured three times, and mean values were used for subsequent analyses. Paternal reproductive investment was operationally estimated as the decrease in male body mass between pre- and post-breeding measurements [44]. In explosive-breeding anurans, this short-term mass loss may reflect the combined effects of mating activity, sperm and seminal fluid release, energetic expenditure, hydration state, and gut-content variation. We therefore treated this variable as a proxy for net breeding-associated male mass change rather than as a direct measure of sperm investment. Changes in female body mass reflect both egg release and energy expenditure during reproductive behaviour, and these components cannot be separated. As a result, female body mass change was not used as a direct measure of reproductive investment. To control for body-size effects on body mass, a body condition index was calculated as the residuals from a linear regression of log-transformed body mass on log-transformed SVL. In subsequent statistical analyses, pre-breeding paternal SVL (PS), paternal body mass (PM), paternal body condition index (paternal BCI), maternal SVL (MS), maternal body mass (MM), and maternal body condition index (maternal BCI) were included as parental size-related variables.

Following oviposition, tissue samples (0.5 mm×1 mm) were collected from the second toe of each adult hind limb for genetic analyses. Toe clipping was performed under anesthesia to minimize injury and reduce potential effects on survival and activity. Genomic DNA was extracted from toe tissue using the TIANamp Genomic DNA Kit (TIANGEN, Beijing, China), dissolved in nuclease-free water, and stored at -20 °C until use. Ten highly polymorphic microsatellite loci (ATM2, ATM21, ATM14, ATM23, ATM22, ATM11, ATM18, ATM32, ATM35, ATM31; Supplementary Table S1) were selected using primers previously developed for the Asiatic toad [45, 46]. PCR amplification was carried out in standard reaction mixtures with fluorescently labeled primers on an ABI 3730xl thermal cycler (Thermo Fisher Scientific, USA). PCR products were separated by capillary electrophoresis, and alleles were scored and genotypes were determined using GeneMarker v.1.3 (SoftGenetics LLC, State College, PA, USA).

Using microsatellite genotypes, we estimated the genetic relatedness coefficient (R) for each parental pair in COANCESTRY v.1.0 [47] with the Lynch & Ritland (1999) [48] estimator [49]. To evaluate whether the marker set met the model assumptions, we used Cervus v.3.0 to calculate the number of alleles per locus (Na), observed and expected heterozygosity (Ho, He), and polymorphic information content (PIC). We then used Arlequin v.3.5 to test for Hardy-Weinberg equilibrium (HWE) and linkage disequilibrium (LD) at each locus. Significance levels for multiple tests were adjusted using Bonferroni correction. Full details of the genetic markers and quality-control procedures are provided in Supplementary Table S1. Across the 20 breeding pairs included in the analyses, pairwise parental relatedness ranged from − 0.2249 to 0.4163 (mean ± SD = -0.0337 ± 0.1467; median = -0.0450).

### Offspring rearing and developmental monitoring

Egg strings were incubated in an environmental chamber at 21 °C with a 12 h light:12 h dark photoperiod (lights on at 0600 h, lights off at 1800 h). Egg strings were placed flat in containers filled with dechlorinated tap water, and the water was changed once per week. Developmental stages of embryos and tadpoles were classified according to Gosner (1960) [50] based on morphological criteria. Once larvae had emerged from the egg jelly and reached stage G25, we randomly selected 30 tadpoles per clutch for subsequent developmental monitoring and released the remaining individuals back into the natal water body. Experimental tadpoles were reared by clutch in plastic tanks (28 cm ×18 cm ×6 cm, L × W × H) containing dechlorinated tap water, with water temperature maintained at 21 ± 2 °C and rearing density kept approximately constant. Water was changed every two days, and tadpoles were fed shrimp eggs twice daily to provide food ad libitum and minimize cannibalism.

We recorded the date on which each tadpole first reached stages G25, G42, and G46. For each clutch and each stage, we calculated the mean number of days and used them as the developmental durations of that clutch at that stage. To avoid mechanical damage from frequent handling, morphological measurements were obtained from photographs. Individuals were photographed under standardized conditions with a ruler (precision 0.1 mm) placed in the frame for spatial calibration. Total length (TL) and body width (BW) at stages G25, G42, and G46 and tibiofibula length (TIL) at G46 were measured from the photographs in Adobe Photoshop v2022 (Adobe Inc, USA). All measurements were recorded to the nearest 0.1 mm.

Offspring development was quantified using three parameters: developmental duration, growth rate, and body size. Developmental duration was measured across three intervals: from fertilization to G25 (G0-G25), from G25 to G42, and from G42 to G46. Growth rate was calculated separately for each interval using the morphological measure most appropriate to that stage. For G0-G25, growth rate was calculated as total length at G25 divided by the duration from fertilization to G25. For G25-G42, growth rate was calculated as the change in total length between G25 and G42 divided by the duration of that interval. For G42-G46, post-metamorphic growth was indexed as tibiofibula length (TIL) at G46 divided by the duration from G42 to G46, because total length is strongly affected by tail resorption during metamorphosis, whereas hindlimb length provides a more reliable measure of post-metamorphic growth and locomotor performance in anurans [51]. Body size was characterized by body length and body width at G25, G42, and G46, together with tibiofibula length at G46.

### Statistical analysis

To quantify assortative mating, we fitted linear models using body size and body condition data from mated males and females. Female traits, including snout-vent length (SVL), body mass, and body condition index (BCI), were used as response variables, and the corresponding male traits were used as predictors to test for positive assortative mating between sexes. In addition, to evaluate among-clutch differences in offspring traits, we performed one-way ANOVAs with clutch identity (pair ID) as a fixed factor for developmental duration, growth rate, and body-size measures at each developmental stage. These analyses tested for significant among-clutch variation in offspring developmental and morphological traits.

We examined the relationships between parental traits and offspring developmental duration using pair-level linear models, because developmental duration was summarized at the clutch level and each parental pair contributed one value for each developmental interval. Therefore, pair identity was not included as a random effect in these models. Stage-specific developmental durations (G0-G25, G25-G42, and G42-G46) were used as response variables and were log-transformed before analysis. To reduce multicollinearity among parental SVL, body mass, and body condition index, we did not include these three body-size-related variables simultaneously in the same model. Instead, we used two complementary predictor sets. The primary models included paternal SVL, paternal body condition index, paternal investment, maternal SVL, maternal body condition index, and the parental relatedness coefficient. A mass-only sensitivity model included paternal body mass, paternal investment, maternal body mass, and the parental relatedness coefficient. All continuous predictors were standardized before model fitting. Model assumptions were assessed using residual diagnostic plots, including residuals versus fitted values and quantile-quantile plots. Multicollinearity among fixed effects was evaluated using variance inflation factors (VIFs) with the R package performance [52].

We used linear mixed-effects models (LMMs) to investigate the relationships between parental traits and offspring growth-related and body-size traits. The stage-specific growth-rate and growth-index variables defined above (G0-G25, G25-G42, and G42-G46), together with body length, body width at each stage, and tibiofibula length after metamorphosis, were used as response variables. Because multiple offspring were measured from each clutch, pair identity was included as a random intercept to account for non-independence among offspring from the same parental pair. For each growth-related and body-size response variable, we fitted models using the same two fixed-effect predictor sets described above: a primary SVL + BCI predictor set and a mass-only sensitivity predictor set. Response variables were log-transformed before analysis, and all continuous predictors were standardized before model fitting. Model assumptions were assessed using residual diagnostic plots, including residuals versus fitted values and quantile-quantile plots. Multicollinearity among fixed effects was evaluated using variance inflation factors (VIFs) with the R package performance [52].

Furthermore, to test whether the effects of parental factors differed among developmental stages, we fitted additional stage-interaction models for growth rate, body length, and body width. For these analyses, repeated measurements of offspring traits across developmental stages were analysed together, and response variables were log-transformed before model fitting. To avoid reintroducing multicollinearity among parental body-size variables, these models were fitted separately for each parental predictor. In each model, developmental stage, one parental predictor, and their interaction were included as fixed effects, with pair identity included as a random intercept to account for non-independence among offspring from the same clutch. These models were fitted using restricted maximum likelihood (REML). The significance of stage-dependent effects was evaluated from the interaction between developmental stage and each parental predictor. Because the primary models tested a priori parental predictors, we interpreted these models using effect sizes, 95% confidence intervals, and exact P values rather than applying a single correction across all response variables. For the exploratory stage-dependent analyses, predictor × stage interaction terms were evaluated using Benjamini-Hochberg false discovery rate correction. All analyses were conducted in R v.4.3.0 [53].

## Results

For assortative mating in parental body size and body condition, we found no statistically significant relationships between the set of paternal traits and maternal snout-vent length or body condition (SVL: *F*_*3, 16*_ = 2.377, *P* = 0.108; BCI: *F*_*3, 16*_ = 2.096, *P* = 0.141). By contrast, the relationship between paternal traits and maternal mass was significant (*F*_*3, 16*_ = 3.693, *P* = 0.034). The simplified linear model showed that paternal mass had a significantly positive effect on maternal mass (*F*_*1, 18*_ = 7.393, *t* = 2.719, *P* = 0.014, *R²* = 0.252). This result indicates positive assortative pairing by body mass, but not by body length or body condition, in this toad species.

One-way ANOVAs showed significant among-clutch differences in developmental duration and growth rate at all three developmental stages (*P* < 0.05). This result indicates substantial among-clutch variation in developmental trajectories. For larval morphology, most traits also varied significantly among clutches (all *P* < 0.05). These traits included total length at each stage, body mass- and body width-related traits, post-metamorphic SVL, and TIL. The only exceptions were body width at stages G42 and G46, which did not differ significantly among clutches (G42: *F*_*18, 201*_ = 1.073, *P* = 0.381; G46: *F*_*18, 289*_ = 1.463, *P* = 0.102).

For maternal traits, the primary SVL + BCI models provided limited evidence for significant maternal effects on offspring developmental duration, growth rate, or morphology. In the primary models, neither maternal snout-vent length (SVL) nor body condition index (BCI) showed significant main effects on stage-specific developmental duration, growth rate, body length, or body width (Supplementary Table S2). In contrast, the mass-only sensitivity models detected a significant association between maternal body mass and tibiofibula length (TIL) at G46, with maternal mass being negatively associated with TIL at this stage (*estimate* = -0.037, *t* = -2.641, *P* = 0.019; Supplementary Table S3).

**Fig. 1:**
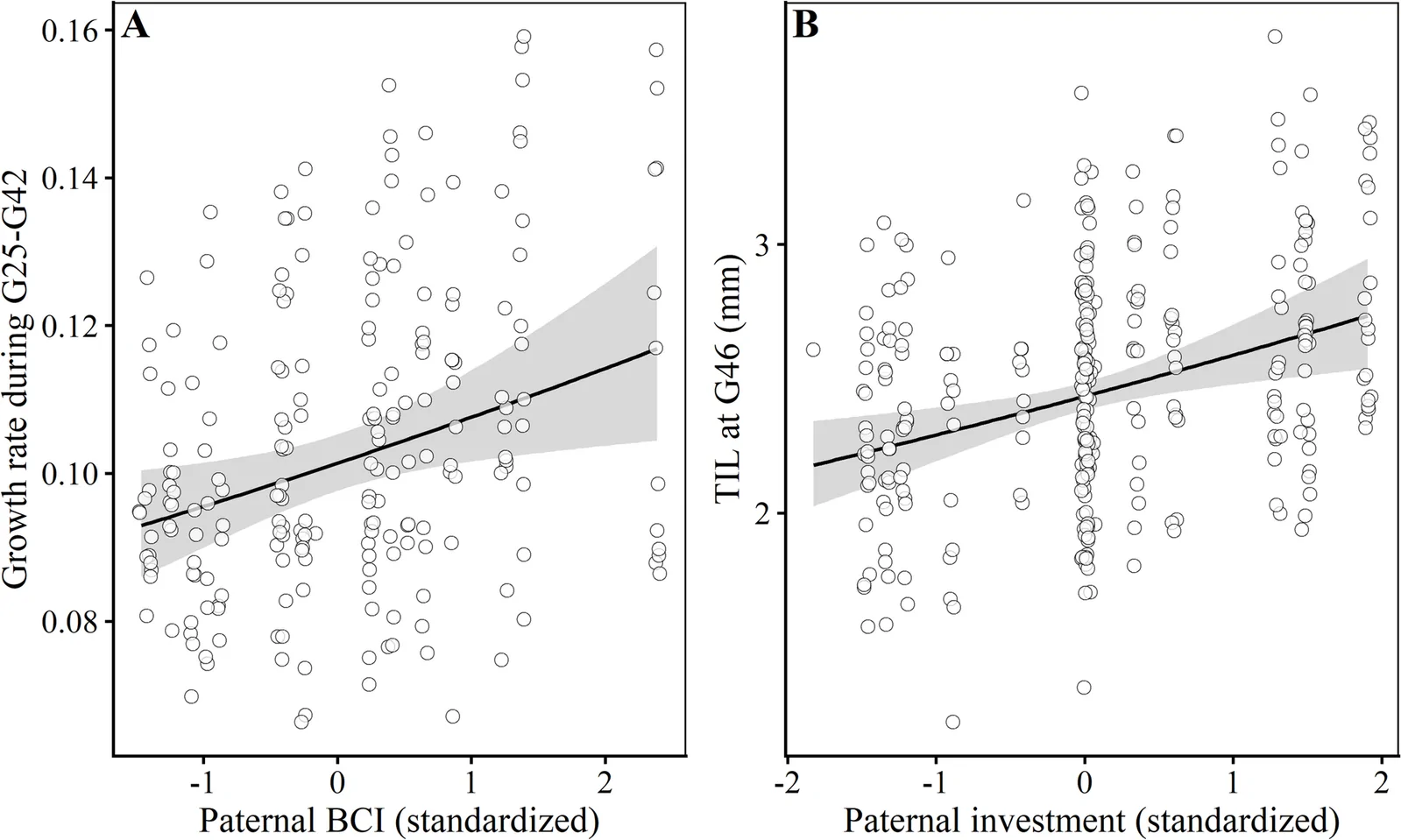
Associations between paternal traits and offspring growth or post-metamorphic morphology in *Bufo gargarizans*. (**A**) Association between paternal body condition index (BCI) and offspring growth rate during the G25-G42 interval, based on the primary SVL + BCI linear mixed-effects model. (**B**) Association between paternal investment and tibiofibula length (TIL) at G46, based on the primary SVL + BCI linear mixed-effects model. Solid lines show fitted values from the multipredictor models with the remaining standardized predictors held at zero, shaded bands indicate 95% confidence intervals, and points show partial residuals

For paternal traits, the primary SVL + BCI models showed that paternal body condition was significantly associated with offspring growth rate during the G25-G42 interval. Specifically, paternal BCI was positively associated with tadpole growth rate at this stage (*estimate* = 0.060, *t* = 2.583, *P* = 0.023; Fig. 1A; Supplementary Table S2). This effect was also supported by the pair-level robustness analysis (*estimate* = 0.061, *t* = 2.765, *P* = 0.016; Supplementary Table S3). Paternal investment was mainly associated with post-metamorphic morphology. In the primary model, paternal investment was positively associated with tibiofibula length (TIL) at G46 (*estimate* = 0.061, *t* = 3.156, *P* = 0.008; Fig. 1B; Supplementary Table S2). In addition, both the pair-level robustness model and the mass-only sensitivity model supported a positive association between paternal investment and body width at G46 (pair-level model: *estimate* = 0.070, *t* = 3.964, *P* = 0.002; mass-only model: *estimate* = 0.037, *t* = 2.233, *P* = 0.041; Supplementary Table S3; Supplementary Figure S2).

**Fig. 2:**
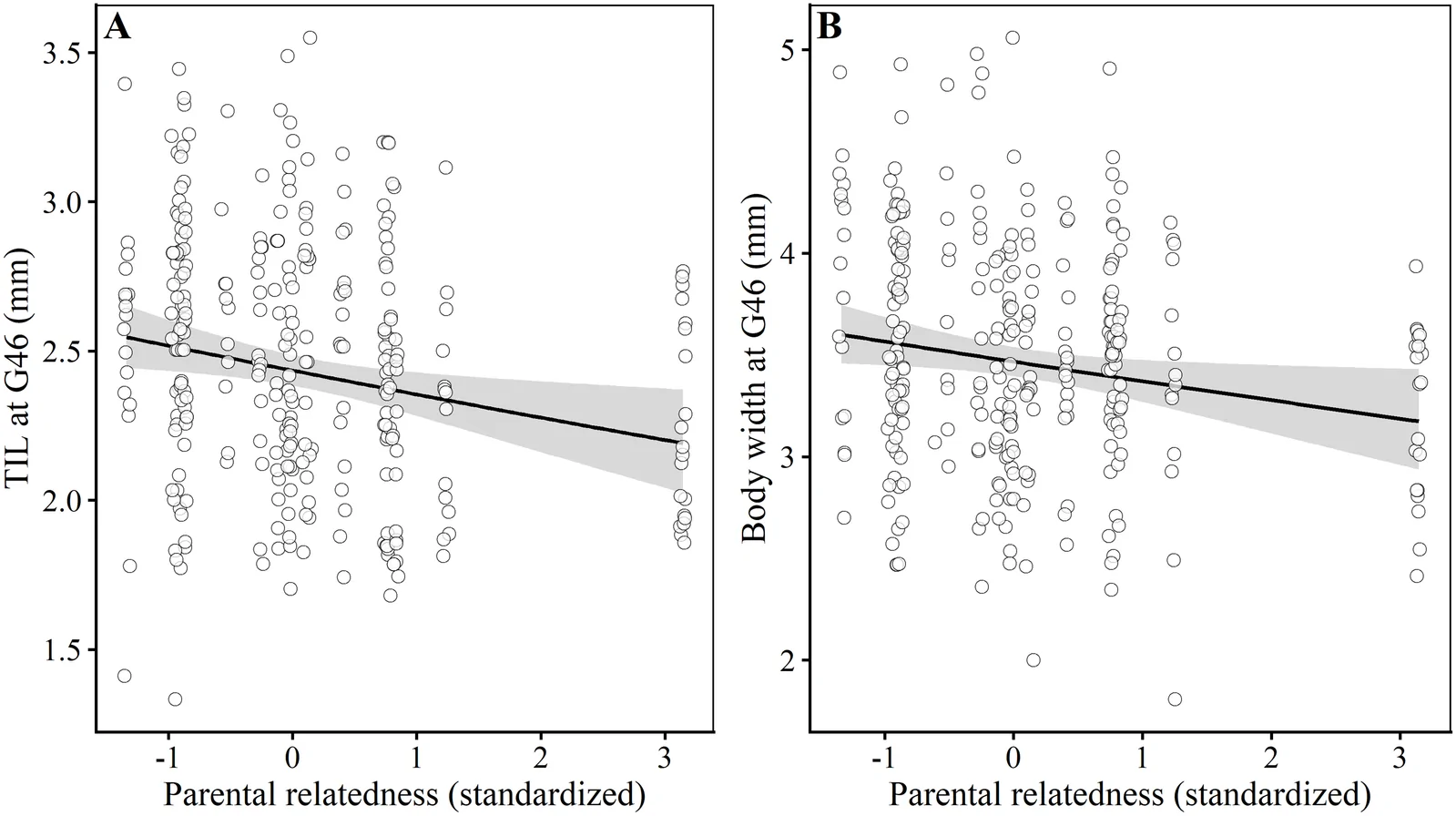
Associations between parental relatedness and post-metamorphic morphology in *Bufo gargarizans*. (**A**) Association between parental relatedness and tibiofibula length (TIL) at G46, based on the primary SVL + BCI linear mixed-effects model. (**B**) Association between parental relatedness and body width at G46, based on the primary SVL + BCI linear mixed-effects model. Solid lines show fitted values from the multipredictor models with the remaining standardized predictors held at zero, shaded bands indicate 95% confidence intervals, and points show partial residuals

For parental relatedness, higher relatedness was negatively associated with post-metamorphic morphology. In the primary SVL + BCI model, higher parental relatedness was associated with shorter tibiofibula length (TIL) at G46 (*estimate* = -0.036, *t* = -2.651, *P* = 0.020; Fig. 2A; Supplementary Table S2) and smaller body width at G46 (*estimate* = -0.030, *t* = -2.261, *P* = 0.042; Fig. 2B; Supplementary Table S2). The negative association between parental relatedness and body width at G46 was also supported by the pair-level robustness analysis (*estimate* = -0.035, *t* = -2.258, *P* = 0.042; Supplementary Table S3). The principal significant associations identified across the main models are summarized in Table 1.

**Table 1:** Summary of the main associations between parental traits and offspring developmental traits in *Bufo gargarizans*

Response trait	Predictor	Model	Estimate	SE	df	Test statistic	*P* value
G46TIL	PI	Primary SVL + BCI	0.061	0.019	13.0	3.156	**0.008**
G46TIL	R	Primary SVL + BCI	-0.036	0.013	13.0	-2.651	**0.020**
G46BW	R	Primary SVL + BCI	-0.030	0.013	13.0	-2.261	**0.042**
G25-42 V	Paternal BCI	Primary SVL + BCI	0.060	0.023	13.0	2.583	**0.023**

Note: Only the principal significant associations are shown. PI, paternal investment; R, parental relatedness coefficient; BCI, body condition index; TIL, tibiofibula length; BW, body width. The primary models included parental SVL and BCI as predictors. Test statistic refers to the coefficient test statistic from the corresponding final model

**Fig. 3:**
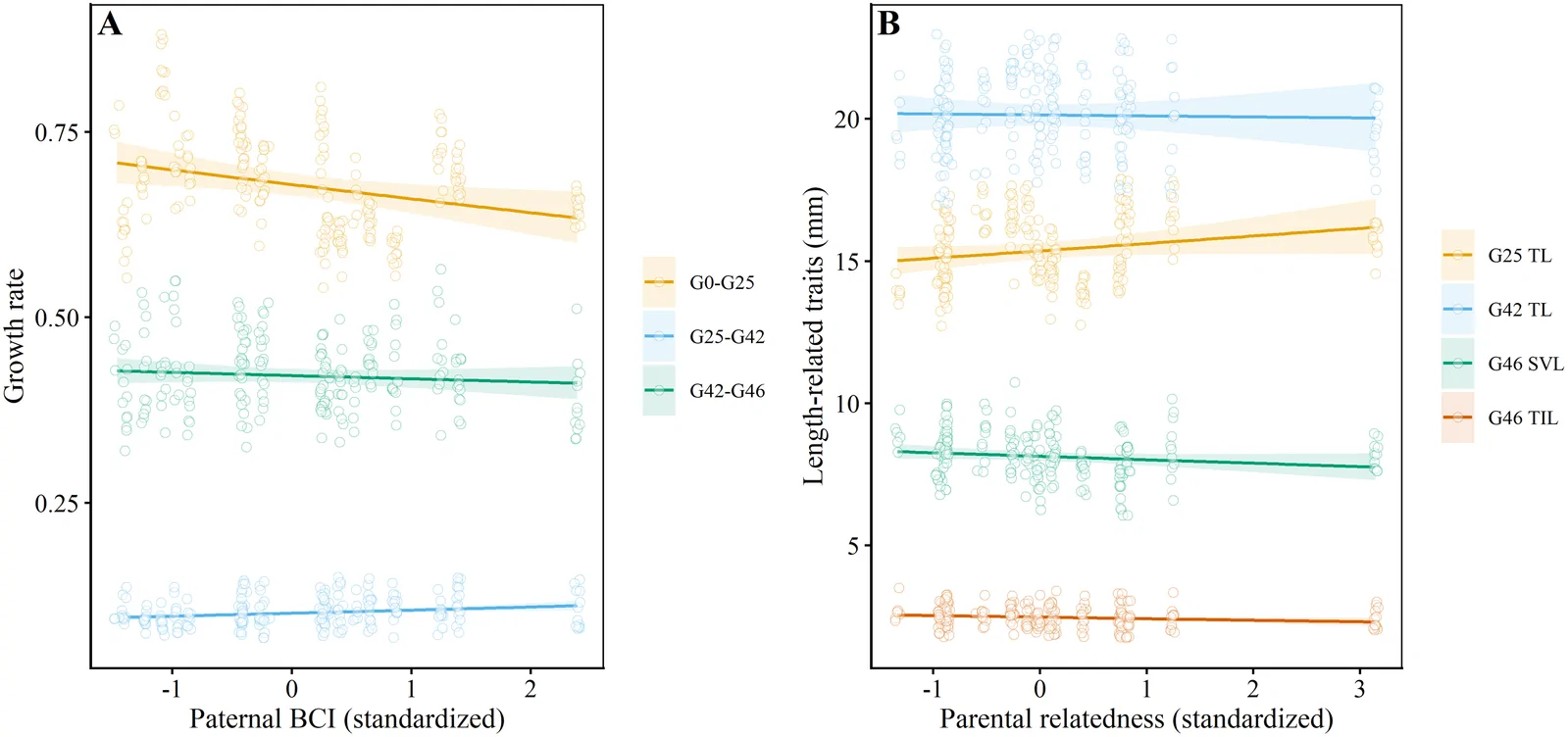
Selected stage-dependent associations between parental predictors and offspring traits in *Bufo gargarizans*. (**A**) Association between paternal body condition index (BCI) and offspring growth rate across developmental stages. (**B**) Association between parental relatedness and length-related offspring traits across developmental stages. Lines show stage-specific fitted values from single-predictor stage-interaction models, shaded bands indicate 95% confidence intervals, and points show observed values

After FDR correction of the predictor × stage interaction terms, two stage-dependent associations remained supported (Fig. 3; Supplementary Table S4). Paternal body condition was associated with offspring growth rate in a stage-dependent manner (paternal BCI × stage: *F*_*2,612*_ = 14.283, *P* < 0.001, *q* < 0.001; Fig. 3A). Parental relatedness also showed a stage-dependent association with length-related offspring traits (R × stage: *F*_*3,822*_ = 5.979, *P* < 0.001, *q* = 0.004; Fig. 3B). Other nominal predictor × stage interactions are reported in Supplementary Table S4 and Supplementary Figure S1.

## Discussion

In this study, we investigated how parental traits, reproductive investment, and parental relatedness were associated with offspring growth and development in the Asiatic toad. We found evidence of positive assortative pairing by body mass, with heavier females tending to pair with heavier males, consistent with previous findings on reproductive behaviour in this species by Luo et al. (2014) [49, 54]. The most consistent associations with offspring developmental and morphological traits involved paternal body condition, paternal investment, and parental relatedness. Specifically, paternal BCI was positively associated with offspring growth rate during the G25-G42 interval, paternal investment was positively associated with post-metamorphic morphology, and higher parental relatedness was associated with reduced length-related morphology around metamorphosis. These results suggest that offspring development in *B. gargarizans* is associated not only with parental phenotype, but also with reproductive investment and parental relatedness. The stage-interaction analyses further indicated that selected associations, particularly those involving paternal BCI and parental relatedness, varied across developmental stages, supporting a dynamic view of parental effects across ontogeny [15, 16, 23]. By explicitly considering parental relatedness and distinguishing among body size, body mass, and body condition, our study provides a useful framework for understanding how parental phenotype and parental relatedness are linked to offspring developmental trajectories.

In amphibians, adult body condition partly reflects internal energy reserves and may influence offspring phenotype through parental effects [55, 56]. Maternal effects are often regarded as important because females can differentially allocate resources, hormones, and other egg components to their offspring, thereby shaping early development and subsequent performance [13, 57]. In the present study, however, maternal snout-vent length (SVL) and maternal body condition showed only limited associations with offspring developmental duration, growth rate, and morphology. This suggests that associations between maternal traits and offspring performance in *B. gargarizans* may be weaker, or more context dependent, than might be expected from studies in which maternal body size or condition represents a major source of parental variation. The mass-only sensitivity analysis suggested a negative association between maternal body mass and tibiofibula length at G46. Although body mass is biologically informative, it can reflect both structural body size and energetic state, making it difficult to determine whether this association was driven by female size, body condition, or other physiological attributes [58]. Similar interpretative challenges have been reported in amphibian studies of maternal effects, where maternal nutritional state, egg provisioning, and offspring performance may be linked in complex or even non-linear ways [12, 23, 59–61]. Thus, although maternal body mass may capture aspects of female physiological state that are not fully represented by SVL or BCI, our results do not support a strong and consistent maternal effect across offspring developmental traits. Nevertheless, the stage-interaction analysis indicated that the association between maternal SVL and offspring growth rate differed among developmental stages. This pattern suggests that maternal size-related effects, if present, may be expressed differently across ontogeny rather than as a single stable main effect [62, 63]. Such stage dependence may arise because larval development involves shifts in morphology, resource use, and energetic demand, which can make parental effects more or less detectable as offspring pass through different developmental transitions [26, 64–66]. Overall, our findings suggest that maternal traits may influence offspring development in a stage-specific or condition-dependent manner, but they were not the strongest or most consistent predictors in this experiment.

Paternal body condition may reflect energetic state and overall physiological quality, and may be linked to offspring performance through both genetic and non-genetic paternal effects [20, 67]. According to the “good genes” hypothesis, male body size or body condition may be positively associated with offspring fitness-related traits, such as growth rate, survival, or competitive ability [68–70]. In the present study, paternal BCI was positively associated with offspring growth rate during the G25-G42 interval, indicating that males in better condition produced offspring with faster mid-larval growth. This result suggests that paternal phenotype may contribute to offspring performance even in externally fertilizing species without post-oviposition parental care [20, 23, 71]. The stage-interaction analysis further indicated that the association between paternal BCI and offspring growth rate differed among developmental stages. This stage dependence may reflect developmental shifts in the relative importance of paternal genetic quality, sperm-mediated effects, and offspring energetic demands. Tadpoles undergo major changes in growth, morphology, and resource use during larval development, and paternal effects may therefore become more apparent during particular developmental windows rather than remaining constant throughout ontogeny [22, 72]. In this study, paternal investment was estimated from male body-mass loss between pre- and post-breeding measurements, which provides a practical index of breeding-associated mass change but does not isolate a single physiological mechanism. This measure may partly reflect sperm or seminal-fluid release, but it may also capture energetic expenditure during amplexus, hydration changes, gut-content variation, or short-term differences in body condition. Thus, the positive associations between this proxy and post-metamorphic morphology should be interpreted as evidence that male breeding-associated mass change is linked to offspring traits, rather than as direct evidence for sperm-mediated paternal effects. Because our experimental design cannot fully distinguish genetic inheritance from non-genetic paternal effects, future studies combining controlled crosses, sperm-quality measurements, and finer-scale genetic analyses would help clarify the mechanisms linking paternal condition to offspring growth. Paternal investment was also positively associated with post-metamorphic morphology, particularly tibiofibula length and body width at G46. These associations suggest that paternal reproductive investment may be linked to offspring size at metamorphosis, a trait with potential consequences for locomotor performance, competitive ability, and early juvenile survival. In explosive-breeding amphibians, males may vary substantially in reproductive investment, and this variation may be associated with fertilization success, embryo quality, or other early developmental processes [73–75]. These patterns suggest that paternal contributions to offspring development may not be limited to fertilization, but may also be reflected in later growth and morphology. Overall, paternal body condition and paternal investment were more consistent predictors than maternal traits in this dataset, highlighting the importance of considering paternal phenotype in studies of amphibian parental effects.

Genetic composition and genotype-specific combinations can influence tadpole growth and development [76, 77], indicating that genetic similarity between breeding individuals may contribute to variation in offspring performance in anurans [78]. In the present study, parental relatedness was negatively associated with post-metamorphic tibiofibula length in *B. gargarizans*. The stage-interaction analysis further showed that the association between parental relatedness and length-related traits varied across development. This pattern suggests that offspring produced by genetically more similar parents may show reduced expression of some length-related morphological traits around metamorphosis [79]. Pairwise relatedness estimates in our sample were generally low and were calculated from a panel of microsatellite markers. Therefore, the observed associations should not be interpreted as direct evidence of close inbreeding. Rather, they suggest that modest variation in parental genetic similarity may be associated with offspring morphological development. Such associations could reflect reduced genetic diversity, increased expression of deleterious alleles, or other genetic compatibility effects, all of which have been proposed to influence offspring performance and developmental stability [79–82]. The stage-dependent association between parental relatedness and length-related morphology further suggests that genetic similarity may not affect all developmental stages equally. This relationship was most evident in later length-related traits, indicating that genetic effects may become more detectable as tadpoles approach metamorphosis. Late larval and metamorphic development involve substantial morphological reorganization, and these stages may be particularly sensitive to genetic or developmental constraints [80, 83]. By contrast, developmental duration showed no clear association with parental relatedness, suggesting that, in this dataset, parental genetic similarity was more closely linked to morphology at metamorphosis than to developmental timing. Developmental rate and final body size may respond differently to genetic and environmental variation, and compensatory growth or resource allocation may partly decouple timing from morphology during larval development [26, 64, 84]. Overall, these results identify parental relatedness as a potential genetic component of offspring phenotypic variation in *B. gargarizans*. They also emphasize that this interpretation should remain cautious, given the limited range of relatedness estimates and the modest number of microsatellite loci.

## Conclusions

This study examined how maternal traits, paternal traits, reproductive investment, and parental relatedness were associated with offspring growth and development in the Asiatic toad. Offspring developmental and morphological traits were most consistently associated with paternal body condition, paternal investment, and parental relatedness. Paternal BCI was associated with mid-stage offspring growth rate, paternal investment was associated with post-metamorphic morphology, and parental relatedness was negatively associated with length-related morphology around metamorphosis. In contrast, maternal SVL and body condition showed limited and mainly stage-dependent associations with offspring traits. These findings suggest that parental effects in *B. gargarizans* are trait-specific and stage-dependent, and that paternal phenotype and parental genetic similarity are important components of offspring developmental variation. By considering parental relatedness alongside parental phenotypic traits, this study provides a broader perspective on how parental phenotype, reproductive investment, and genetic similarity are linked to offspring development. Several limitations should be acknowledged. First, we did not explicitly account for egg size, clutch quality, or hatching success, which may strongly affect early developmental stages and limit a more comprehensive assessment of early parental effects. Second, parental traits are themselves influenced by environmental conditions, and part of the variation in offspring traits is likely to be genetically based. With the current experimental design, genetic and environmental contributions cannot be fully separated. Future studies combining controlled crosses, finer-scale genetic analyses, sperm-quality measurements, and experimental manipulation of parental condition would help clarify the mechanisms by which parental traits and parental genetic similarity jointly shape offspring development. Overall, this study contributes to the framework of parental effects in amphibians and highlights the importance of integrating paternal phenotype and parental relatedness when assessing offspring developmental and morphological variation.

## Supplementary Information

Below is the link to the electronic supplementary material.

Supplementary Material 1

Supplementary Material 2

## Data Availability

The datasets used and/or analyzed during the current study are available from the corresponding author on reasonable request.

## Abbreviations

SVL: Snout–vent length
PS: Paternal snout-vent length
PM: Paternal body mass
BCI: Body condition index
MS: Maternal snout–vent length
MM: Maternal body mass
DNA: Deoxyribonucleic acid
PCR: Polymerase chain reaction
ABI: Applied biosystems
Na: Number of alleles per locus
Ho: Observed heterozygosity
He: Expected heterozygosity
PIC: Polymorphic information content
HWE: Hardy-Weinberg equilibrium
LD: Linkage disequilibrium
TL: Total length
BW: Body width
TIL: Tibiofibula length
ANOVA: Analysis of variance
VIFs: Variance inflation factors
LMMs: Linear mixed-effects models
REML: Restricted maximum likelihood
