# Supplementary material for: Parental traits, reproductive investment, and genetic relatedness shape offspring development and morphology in an explosive-breeding toad
Source: Front Zool. 2026 Aug 11;23:39. doi: 10.1186/s12983-026-00627-0 (PMC13750053; doi:10.1186/s12983-026-00627-0)
Supplement: Supplementary file 1 [file 12983_2026_627_MOESM1_ESM.docx]

**Supplementary Information**

**Additional file 1**

**Table S1**

**Table S1** Microsatellite loci, primer sequences, fluorescent labels, annealing temperatures, and allele sizes for genotyping relatedness between males and females of *Bufo gargarizans*.

| **Locus** | **Primer sequence (5′–3′)** | **Repeat motif** | **Fluorescent label** | **Annealing temperature (°C)** | **Allele size range (bp)** |
| --- | --- | --- | --- | --- | --- |
| ATM2 | F: ATGGGTTATGGTTGGGAAGG  R: GATTGCTGTGGACCAGGTGT | (TTTA)_6_ | FAM | 58.0 | 209–237 |
| ATM21 | F: AGGGCAAGTTCTGATCAACG  R: GATACGGTCGTGAATGAGGC | (TA)_11_ | HEX | 57.0 | 282–312 |
| ATM14 | F: CTTTCGTTTATGGCCCTTCC  R: GTGTGAGGATCCAGGCTGAC | (GA)_10_ | TAMRA | 58.0 | 376–396 |
| ATM23 | F: GCTCAGCACAGCATAGGAAA  R: ACCAGTACACAGACAGGCCG | (AG)_10_ | FAM | 58.0 | 68–80 |
| ATM22 | F: CATTAATTTGCCGAGGCTTG  R: CTAACGCAGCACCAGGCTAT | (GT)_8_ | HEX | 57.0 | 186–200 |
| ATM11 | F: TTAGAGCAGCGGGTTACGTC  R: CCTCGTTGTGGGTACAGGAG | (TTTG)_6_ | FAM | 58.0 | 301–317 |
| ATM18 | F: GGCAGGAGACACCTGACTGT  R: ATGGCCGATGTGACAAGTCT | (TG)_10_ | FAM | 60.5 | 105–121 |
| ATM32 | F: ATCTCAGCCTGCTCCAGTTG  R: TGGTGGTACAGGAACACCCT | (TG)_10_ | TAMRA | 56.0 | 121–127 |
| ATM35 | F: CATGGCCTGCAGTATCAGTG  R: CATTACTGCTGCTCCCTGCT | (ACC)_12_ | TAMRA | 62.0 | 165–198 |
| ATM31 | F: CAAACAATGCTTTCCCGTTT  R: TAACGGTTCCATGAATTGGC | (AG)_11_ | TAMRA | 58.0 | 300–322 |

Note: All PCRs were performed in 10 μL reaction volumes containing approximately 1 μL genomic DNA, 6 μL Premix Taq (TaKaRa), 0.6 μL primer mix (0.3 μL of each primer) and nuclease-free water to volume. Cycling conditions were: 95 °C for 5 min; 35 cycles of 94 °C for 30 s, locus-specific annealing temperature (Ta, see table) for 30 s, and 72 °C for 30 s; followed by a final extension at 72 °C for 5 min.

**Table S2**

Supplementary Table S2. Fixed-effect estimates from the primary SVL + BCI models.

| Model | Response | Predictor | Estimate | SE | df | Test statistic | P value | 95% CI |
| --- | --- | --- | --- | --- | --- | --- | --- | --- |
| Duration primary SVL+BCI | G0-25DD | PS | 0.037 | 0.060 |  | 0.617 | 0.548 | [-0.092, 0.166] |
| Duration primary SVL+BCI | G0-25DD | Paternal BCI | 0.062 | 0.053 |  | 1.158 | 0.268 | [-0.053, 0.177] |
| Duration primary SVL+BCI | G0-25DD | PI | 0.001 | 0.067 |  | 0.011 | 0.991 | [-0.143, 0.145] |
| Duration primary SVL+BCI | G0-25DD | MS | -0.072 | 0.060 |  | -1.208 | 0.248 | [-0.201, 0.057] |
| Duration primary SVL+BCI | G0-25DD | Maternal BCI | -0.049 | 0.051 |  | -0.958 | 0.356 | [-0.158, 0.061] |
| Duration primary SVL+BCI | G0-25DD | R | 0.061 | 0.058 |  | 1.047 | 0.314 | [-0.065, 0.186] |
| Duration primary SVL+BCI | G25-42DD | PS | -0.001 | 0.050 |  | -0.019 | 0.985 | [-0.108, 0.106] |
| Duration primary SVL+BCI | G25-42DD | Paternal BCI | -0.041 | 0.044 |  | -0.916 | 0.376 | [-0.136, 0.055] |
| Duration primary SVL+BCI | G25-42DD | PI | 0.058 | 0.055 |  | 1.042 | 0.316 | [-0.062, 0.177] |
| Duration primary SVL+BCI | G25-42DD | MS | -0.006 | 0.050 |  | -0.119 | 0.907 | [-0.113, 0.101] |
| Duration primary SVL+BCI | G25-42DD | Maternal BCI | -0.077 | 0.042 |  | -1.829 | 0.090 | [-0.168, 0.014] |
| Duration primary SVL+BCI | G25-42DD | R | 0.000 | 0.048 |  | 0.006 | 0.995 | [-0.104, 0.104] |
| Duration primary SVL+BCI | G42-46DD | PS | -0.016 | 0.030 |  | -0.536 | 0.601 | [-0.080, 0.048] |
| Duration primary SVL+BCI | G42-46DD | Paternal BCI | -0.004 | 0.027 |  | -0.139 | 0.892 | [-0.061, 0.054] |
| Duration primary SVL+BCI | G42-46DD | PI | 0.035 | 0.033 |  | 1.052 | 0.312 | [-0.037, 0.107] |
| Duration primary SVL+BCI | G42-46DD | MS | -0.008 | 0.030 |  | -0.265 | 0.796 | [-0.072, 0.057] |
| Duration primary SVL+BCI | G42-46DD | Maternal BCI | -0.018 | 0.025 |  | -0.720 | 0.485 | [-0.073, 0.037] |
| Duration primary SVL+BCI | G42-46DD | R | -0.004 | 0.029 |  | -0.152 | 0.882 | [-0.067, 0.058] |
| Rate primary SVL+BCI | G0-25V | PS | 0.003 | 0.026 | 13 | 0.122 | 0.905 | [-0.047, 0.054] |
| Rate primary SVL+BCI | G0-25V | Paternal BCI | -0.041 | 0.023 | 13 | -1.790 | 0.097 | [-0.086, 0.004] |
| Rate primary SVL+BCI | G0-25V | PI | 0.015 | 0.029 | 13 | 0.522 | 0.610 | [-0.041, 0.071] |
| Rate primary SVL+BCI | G0-25V | MS | 0.013 | 0.026 | 13 | 0.518 | 0.613 | [-0.037, 0.064] |
| Rate primary SVL+BCI | G0-25V | Maternal BCI | 0.018 | 0.022 | 13 | 0.818 | 0.428 | [-0.025, 0.061] |
| Rate primary SVL+BCI | G0-25V | R | -0.007 | 0.025 | 13 | -0.287 | 0.778 | [-0.056, 0.042] |
| Rate primary SVL+BCI | G25-42V | PS | 0.022 | 0.027 | 13 | 0.827 | 0.423 | [-0.031, 0.075] |
| **Rate primary SVL+BCI** | **G25-42V** | **Paternal BCI** | **0.060** | **0.023** | **13** | **2.583** | **0.023** | **[0.014, 0.105]** |
| Rate primary SVL+BCI | G25-42V | PI | -0.019 | 0.032 | 13 | -0.601 | 0.558 | [-0.082, 0.044] |
| Rate primary SVL+BCI | G25-42V | MS | -0.014 | 0.025 | 13 | -0.544 | 0.596 | [-0.063, 0.036] |
| Rate primary SVL+BCI | G25-42V | Maternal BCI | -0.024 | 0.023 | 13 | -1.053 | 0.311 | [-0.069, 0.021] |
| Rate primary SVL+BCI | G25-42V | R | -0.004 | 0.024 | 13 | -0.170 | 0.868 | [-0.051, 0.043] |
| Rate primary SVL+BCI | G42-46V | PS | 0.011 | 0.021 | 13 | 0.534 | 0.603 | [-0.030, 0.052] |
| Rate primary SVL+BCI | G42-46V | Paternal BCI | -0.021 | 0.017 | 13 | -1.219 | 0.245 | [-0.055, 0.013] |
| Rate primary SVL+BCI | G42-46V | PI | 0.007 | 0.024 | 13 | 0.280 | 0.784 | [-0.040, 0.053] |
| Rate primary SVL+BCI | G42-46V | MS | 0.001 | 0.019 | 13 | 0.073 | 0.943 | [-0.035, 0.038] |
| Rate primary SVL+BCI | G42-46V | Maternal BCI | 0.021 | 0.017 | 13 | 1.214 | 0.246 | [-0.013, 0.054] |
| Rate primary SVL+BCI | G42-46V | R | -0.015 | 0.018 | 13 | -0.795 | 0.441 | [-0.051, 0.021] |
| Length primary SVL+BCI | G25TL | PS | 0.009 | 0.020 | 13 | 0.445 | 0.664 | [-0.030, 0.048] |
| Length primary SVL+BCI | G25TL | Paternal BCI | -0.020 | 0.018 | 13 | -1.099 | 0.292 | [-0.055, 0.015] |
| Length primary SVL+BCI | G25TL | PI | -0.004 | 0.022 | 13 | -0.174 | 0.864 | [-0.048, 0.040] |
| Length primary SVL+BCI | G25TL | MS | 0.007 | 0.020 | 13 | 0.369 | 0.718 | [-0.032, 0.047] |
| Length primary SVL+BCI | G25TL | Maternal BCI | 0.001 | 0.017 | 13 | 0.078 | 0.939 | [-0.032, 0.035] |
| Length primary SVL+BCI | G25TL | R | 0.015 | 0.020 | 13 | 0.778 | 0.451 | [-0.023, 0.053] |
| Length primary SVL+BCI | G42TL | PS | 0.005 | 0.009 | 13 | 0.508 | 0.620 | [-0.013, 0.023] |
| Length primary SVL+BCI | G42TL | Paternal BCI | 0.002 | 0.008 | 13 | 0.202 | 0.843 | [-0.014, 0.017] |
| Length primary SVL+BCI | G42TL | PI | -0.008 | 0.011 | 13 | -0.735 | 0.475 | [-0.029, 0.013] |
| Length primary SVL+BCI | G42TL | MS | 0.016 | 0.009 | 13 | 1.751 | 0.103 | [-0.002, 0.033] |
| Length primary SVL+BCI | G42TL | Maternal BCI | 0.004 | 0.008 | 13 | 0.492 | 0.631 | [-0.011, 0.019] |
| Length primary SVL+BCI | G42TL | R | -0.007 | 0.008 | 13 | -0.863 | 0.404 | [-0.023, 0.009] |
| Length primary SVL+BCI | G46SVL | PS | -0.003 | 0.017 | 13 | -0.148 | 0.885 | [-0.037, 0.031] |
| Length primary SVL+BCI | G46SVL | Paternal BCI | -0.017 | 0.015 | 13 | -1.108 | 0.288 | [-0.046, 0.013] |
| Length primary SVL+BCI | G46SVL | PI | 0.027 | 0.021 | 13 | 1.270 | 0.226 | [-0.015, 0.069] |
| Length primary SVL+BCI | G46SVL | MS | -0.012 | 0.016 | 13 | -0.748 | 0.468 | [-0.044, 0.020] |
| Length primary SVL+BCI | G46SVL | Maternal BCI | 0.005 | 0.014 | 13 | 0.364 | 0.722 | [-0.023, 0.033] |
| Length primary SVL+BCI | G46SVL | R | -0.021 | 0.016 | 13 | -1.312 | 0.212 | [-0.054, 0.011] |
| Length primary SVL+BCI | G46TIL | PS | -0.003 | 0.014 | 13 | -0.209 | 0.838 | [-0.031, 0.025] |
| Length primary SVL+BCI | G46TIL | Paternal BCI | -0.006 | 0.014 | 13 | -0.457 | 0.655 | [-0.033, 0.021] |
| **Length primary SVL+BCI** | **G46TIL** | **PI** | **0.061** | **0.019** | **13** | **3.156** | **0.008** | **[0.023, 0.099]** |
| Length primary SVL+BCI | G46TIL | MS | -0.014 | 0.016 | 13 | -0.880 | 0.395 | [-0.044, 0.017] |
| Length primary SVL+BCI | G46TIL | Maternal BCI | -0.026 | 0.014 | 13 | -1.873 | 0.084 | [-0.052, 0.001] |
| **Length primary SVL+BCI** | **G46TIL** | **R** | **-0.036** | **0.013** | **13** | **-2.651** | **0.020** | **[-0.062, -0.009]** |
| Width primary SVL+BCI | G25BW | PS | 0.026 | 0.024 | 13 | 1.079 | 0.300 | [-0.022, 0.074] |
| Width primary SVL+BCI | G25BW | Paternal BCI | -0.025 | 0.022 | 13 | -1.144 | 0.273 | [-0.068, 0.018] |
| Width primary SVL+BCI | G25BW | PI | 0.003 | 0.027 | 13 | 0.096 | 0.925 | [-0.051, 0.056] |
| Width primary SVL+BCI | G25BW | MS | -0.012 | 0.025 | 13 | -0.507 | 0.621 | [-0.061, 0.036] |
| Width primary SVL+BCI | G25BW | Maternal BCI | 0.004 | 0.021 | 13 | 0.188 | 0.854 | [-0.037, 0.045] |
| Width primary SVL+BCI | G25BW | R | 0.012 | 0.024 | 13 | 0.501 | 0.624 | [-0.035, 0.059] |
| Width primary SVL+BCI | G42BW | PS | 0.012 | 0.013 | 13 | 0.930 | 0.370 | [-0.013, 0.037] |
| Width primary SVL+BCI | G42BW | Paternal BCI | -0.005 | 0.011 | 13 | -0.497 | 0.628 | [-0.027, 0.016] |
| Width primary SVL+BCI | G42BW | PI | -0.023 | 0.015 | 13 | -1.513 | 0.154 | [-0.054, 0.007] |
| Width primary SVL+BCI | G42BW | MS | 0.017 | 0.013 | 13 | 1.310 | 0.213 | [-0.008, 0.042] |
| Width primary SVL+BCI | G42BW | Maternal BCI | -0.014 | 0.011 | 13 | -1.248 | 0.234 | [-0.035, 0.008] |
| Width primary SVL+BCI | G42BW | R | 0.019 | 0.012 | 13 | 1.618 | 0.130 | [-0.004, 0.041] |
| Width primary SVL+BCI | G46BW | PS | -0.002 | 0.014 | 13 | -0.173 | 0.866 | [-0.030, 0.025] |
| Width primary SVL+BCI | G46BW | Paternal BCI | -0.003 | 0.013 | 13 | -0.215 | 0.833 | [-0.029, 0.023] |
| Width primary SVL+BCI | G46BW | PI | 0.038 | 0.019 | 13 | 2.028 | 0.064 | [0.001, 0.076] |
| Width primary SVL+BCI | G46BW | MS | -0.002 | 0.015 | 13 | -0.140 | 0.891 | [-0.032, 0.028] |
| Width primary SVL+BCI | G46BW | Maternal BCI | 0.000 | 0.013 | 13 | 0.016 | 0.987 | [-0.026, 0.026] |
| **Width primary SVL+BCI** | **G46BW** | **R** | **-0.030** | **0.013** | **13** | **-2.261** | **0.042** | **[-0.056, -0.004]** |

Note: Primary models included parental SVL and BCI as predictors, together with paternal investment and parental relatedness. PS, paternal SVL; MS, maternal SVL; BCI, body condition index; PI, paternal investment; R, parental relatedness coefficient; DD, developmental duration; V, growth rate; TIL, tibiofibula length; BW, body width. Confidence intervals are shown for fixed-effect estimates. Degrees of freedom are reported where applicable.

**Table S3**

Supplementary Table S3. Fixed-effect estimates from the mass-only sensitivity models and pair-level robustness analyses.

| Model | Response | Predictor | Estimate | SE | df | Test statistic | P value | 95% CI |
| --- | --- | --- | --- | --- | --- | --- | --- | --- |
| Duration mass sensitivity | G0-25DD | PM | 0.030 | 0.058 |  | 0.516 | 0.613 | [-0.094, 0.154] |
| Duration mass sensitivity | G0-25DD | PI | 0.010 | 0.064 |  | 0.157 | 0.877 | [-0.127, 0.148] |
| Duration mass sensitivity | G0-25DD | MM | -0.053 | 0.055 |  | -0.954 | 0.355 | [-0.170, 0.065] |
| Duration mass sensitivity | G0-25DD | R | 0.041 | 0.054 |  | 0.759 | 0.459 | [-0.074, 0.156] |
| Duration mass sensitivity | G25-42DD | PM | 0.010 | 0.049 |  | 0.204 | 0.841 | [-0.094, 0.114] |
| Duration mass sensitivity | G25-42DD | PI | 0.050 | 0.054 |  | 0.914 | 0.375 | [-0.066, 0.166] |
| Duration mass sensitivity | G25-42DD | MM | -0.090 | 0.046 |  | -1.932 | 0.072 | [-0.189, 0.009] |
| Duration mass sensitivity | G25-42DD | R | 0.019 | 0.046 |  | 0.410 | 0.688 | [-0.079, 0.116] |
| Duration mass sensitivity | G42-46DD | PM | -0.015 | 0.028 |  | -0.562 | 0.582 | [-0.074, 0.043] |
| Duration mass sensitivity | G42-46DD | PI | 0.036 | 0.031 |  | 1.176 | 0.258 | [-0.029, 0.101] |
| Duration mass sensitivity | G42-46DD | MM | -0.021 | 0.026 |  | -0.805 | 0.433 | [-0.077, 0.035] |
| Duration mass sensitivity | G42-46DD | R | -0.003 | 0.026 |  | -0.132 | 0.897 | [-0.058, 0.051] |
| Rate mass sensitivity | G0-25V | PM | 0.006 | 0.027 | 15 | 0.228 | 0.823 | [-0.047, 0.059] |
| Rate mass sensitivity | G0-25V | PI | 0.003 | 0.030 | 15 | 0.093 | 0.927 | [-0.056, 0.062] |
| Rate mass sensitivity | G0-25V | MM | 0.007 | 0.026 | 15 | 0.279 | 0.784 | [-0.043, 0.057] |
| Rate mass sensitivity | G0-25V | R | 0.002 | 0.025 | 15 | 0.075 | 0.941 | [-0.048, 0.051] |
| Rate mass sensitivity | G25-42V | PM | 0.013 | 0.031 | 15 | 0.428 | 0.675 | [-0.048, 0.074] |
| Rate mass sensitivity | G25-42V | PI | -0.000 | 0.035 | 15 | -0.001 | 0.999 | [-0.069, 0.069] |
| Rate mass sensitivity | G25-42V | MM | -0.008 | 0.030 | 15 | -0.280 | 0.783 | [-0.067, 0.050] |
| Rate mass sensitivity | G25-42V | R | -0.020 | 0.027 | 15 | -0.726 | 0.479 | [-0.072, 0.033] |
| Rate mass sensitivity | G42-46V | PM | 0.013 | 0.021 | 15 | 0.644 | 0.529 | [-0.027, 0.054] |
| Rate mass sensitivity | G42-46V | PI | -0.003 | 0.023 | 15 | -0.123 | 0.904 | [-0.049, 0.043] |
| Rate mass sensitivity | G42-46V | MM | 0.011 | 0.020 | 15 | 0.536 | 0.600 | [-0.028, 0.049] |
| Rate mass sensitivity | G42-46V | R | -0.010 | 0.018 | 15 | -0.537 | 0.599 | [-0.044, 0.025] |
| Length mass sensitivity | G25TL | PM | 0.013 | 0.020 | 15 | 0.680 | 0.507 | [-0.025, 0.052] |
| Length mass sensitivity | G25TL | PI | -0.011 | 0.022 | 15 | -0.497 | 0.626 | [-0.054, 0.032] |
| Length mass sensitivity | G25TL | MM | -0.004 | 0.019 | 15 | -0.207 | 0.839 | [-0.040, 0.033] |
| Length mass sensitivity | G25TL | R | 0.021 | 0.018 | 15 | 1.163 | 0.263 | [-0.015, 0.057] |
| Length mass sensitivity | G42TL | PM | 0.008 | 0.009 | 15 | 0.845 | 0.412 | [-0.010, 0.026] |
| Length mass sensitivity | G42TL | PI | -0.006 | 0.011 | 15 | -0.508 | 0.619 | [-0.027, 0.016] |
| Length mass sensitivity | G42TL | MM | 0.009 | 0.009 | 15 | 0.979 | 0.343 | [-0.009, 0.026] |
| Length mass sensitivity | G42TL | R | -0.005 | 0.008 | 15 | -0.589 | 0.565 | [-0.021, 0.011] |
| Length mass sensitivity | G46SVL | PM | -0.005 | 0.017 | 15 | -0.305 | 0.765 | [-0.039, 0.029] |
| Length mass sensitivity | G46SVL | PI | 0.021 | 0.021 | 15 | 1.017 | 0.325 | [-0.019, 0.061] |
| Length mass sensitivity | G46SVL | MM | -0.008 | 0.017 | 15 | -0.467 | 0.647 | [-0.041, 0.025] |
| Length mass sensitivity | G46SVL | R | -0.019 | 0.016 | 15 | -1.223 | 0.240 | [-0.049, 0.011] |
| Length mass sensitivity | G46TIL | PM | -0.003 | 0.014 | 15 | -0.232 | 0.820 | [-0.031, 0.024] |
| **Length mass sensitivity** | **G46TIL** | **PI** | **0.062** | **0.017** | **15** | **3.624** | **0.002** | **[0.029, 0.096]** |
| **Length mass sensitivity** | **G46TIL** | **MM** | **-0.037** | **0.014** | **15** | **-2.641** | **0.019** | **[-0.064, -0.010]** |
| **Length mass sensitivity** | **G46TIL** | **R** | **-0.034** | **0.012** | **15** | **-2.800** | **0.013** | **[-0.058, -0.010]** |
| Width mass sensitivity | G25BW | PM | 0.029 | 0.025 | 15 | 1.153 | 0.267 | [-0.020, 0.077] |
| Width mass sensitivity | G25BW | PI | -0.009 | 0.028 | 15 | -0.317 | 0.755 | [-0.063, 0.045] |
| Width mass sensitivity | G25BW | MM | -0.014 | 0.024 | 15 | -0.581 | 0.570 | [-0.060, 0.032] |
| Width mass sensitivity | G25BW | R | 0.016 | 0.023 | 15 | 0.677 | 0.509 | [-0.030, 0.061] |
| Width mass sensitivity | G42BW | PM | 0.018 | 0.013 | 15 | 1.427 | 0.174 | [-0.007, 0.043] |
| Width mass sensitivity | G42BW | PI | -0.019 | 0.015 | 15 | -1.261 | 0.226 | [-0.049, 0.011] |
| Width mass sensitivity | G42BW | MM | -0.011 | 0.012 | 15 | -0.870 | 0.398 | [-0.035, 0.013] |
| Width mass sensitivity | G42BW | R | 0.023 | 0.011 | 15 | 2.039 | 0.059 | [0.001, 0.046] |
| Width mass sensitivity | G46BW | PM | -0.001 | 0.014 | 15 | -0.101 | 0.921 | [-0.028, 0.025] |
| **Width mass sensitivity** | **G46BW** | **PI** | **0.037** | **0.016** | **15** | **2.233** | **0.041** | **[0.004, 0.069]** |
| Width mass sensitivity | G46BW | MM | -0.003 | 0.013 | 15 | -0.197 | 0.847 | [-0.029, 0.024] |
| **Width mass sensitivity** | **G46BW** | **R** | **-0.029** | **0.012** | **15** | **-2.479** | **0.026** | **[-0.052, -0.006]** |
| Rate pair robustness | G0-25V | PS | 0.003 | 0.026 |  | 0.118 | 0.908 | [-0.054, 0.060] |
| Rate pair robustness | G0-25V | Paternal BCI | -0.043 | 0.024 |  | -1.817 | 0.092 | [-0.094, 0.008] |
| Rate pair robustness | G0-25V | PI | 0.016 | 0.029 |  | 0.537 | 0.600 | [-0.048, 0.079] |
| Rate pair robustness | G0-25V | MS | 0.013 | 0.026 |  | 0.495 | 0.629 | [-0.044, 0.070] |
| Rate pair robustness | G0-25V | Maternal BCI | 0.019 | 0.022 |  | 0.840 | 0.416 | [-0.030, 0.067] |
| Rate pair robustness | G0-25V | R | -0.008 | 0.026 |  | -0.300 | 0.769 | [-0.063, 0.048] |
| Rate pair robustness | G25-42V | PS | 0.012 | 0.025 |  | 0.502 | 0.624 | [-0.041, 0.066] |
| **Rate pair robustness** | **G25-42V** | **Paternal BCI** | **0.061** | **0.022** |  | **2.765** | **0.016** | **[0.013, 0.109]** |
| Rate pair robustness | G25-42V | PI | 0.001 | 0.028 |  | 0.028 | 0.978 | [-0.059, 0.060] |
| Rate pair robustness | G25-42V | MS | -0.020 | 0.025 |  | -0.807 | 0.434 | [-0.073, 0.033] |
| Rate pair robustness | G25-42V | Maternal BCI | -0.031 | 0.021 |  | -1.464 | 0.167 | [-0.076, 0.015] |
| Rate pair robustness | G25-42V | R | -0.011 | 0.024 |  | -0.479 | 0.640 | [-0.063, 0.040] |
| Rate pair robustness | G42-46V | PS | 0.017 | 0.023 |  | 0.753 | 0.465 | [-0.032, 0.067] |
| Rate pair robustness | G42-46V | Paternal BCI | -0.022 | 0.020 |  | -1.068 | 0.305 | [-0.066, 0.022] |
| Rate pair robustness | G42-46V | PI | -0.020 | 0.025 |  | -0.766 | 0.457 | [-0.075, 0.036] |
| Rate pair robustness | G42-46V | MS | 0.014 | 0.023 |  | 0.605 | 0.555 | [-0.036, 0.063] |
| Rate pair robustness | G42-46V | Maternal BCI | 0.032 | 0.019 |  | 1.671 | 0.119 | [-0.010, 0.074] |
| Rate pair robustness | G42-46V | R | -0.011 | 0.022 |  | -0.510 | 0.619 | [-0.059, 0.037] |
| Length pair robustness | G25TL | PS | 0.009 | 0.021 |  | 0.434 | 0.671 | [-0.036, 0.054] |
| Length pair robustness | G25TL | Paternal BCI | -0.021 | 0.018 |  | -1.159 | 0.267 | [-0.061, 0.018] |
| Length pair robustness | G25TL | PI | -0.003 | 0.023 |  | -0.132 | 0.897 | [-0.053, 0.047] |
| Length pair robustness | G25TL | MS | 0.006 | 0.021 |  | 0.305 | 0.766 | [-0.038, 0.051] |
| Length pair robustness | G25TL | Maternal BCI | 0.003 | 0.018 |  | 0.144 | 0.888 | [-0.035, 0.040] |
| Length pair robustness | G25TL | R | 0.015 | 0.020 |  | 0.747 | 0.468 | [-0.028, 0.058] |
| Length pair robustness | G42TL | PS | -0.003 | 0.013 |  | -0.211 | 0.836 | [-0.031, 0.025] |
| Length pair robustness | G42TL | Paternal BCI | -0.000 | 0.012 |  | -0.029 | 0.977 | [-0.025, 0.025] |
| Length pair robustness | G42TL | PI | 0.015 | 0.014 |  | 1.024 | 0.324 | [-0.016, 0.046] |
| Length pair robustness | G42TL | MS | 0.006 | 0.013 |  | 0.446 | 0.663 | [-0.022, 0.034] |
| Length pair robustness | G42TL | Maternal BCI | -0.003 | 0.011 |  | -0.303 | 0.767 | [-0.027, 0.020] |
| Length pair robustness | G42TL | R | -0.008 | 0.013 |  | -0.655 | 0.524 | [-0.035, 0.019] |
| Length pair robustness | G46SVL | PS | -0.003 | 0.016 |  | -0.163 | 0.873 | [-0.037, 0.032] |
| Length pair robustness | G46SVL | Paternal BCI | -0.015 | 0.014 |  | -1.072 | 0.303 | [-0.046, 0.015] |
| Length pair robustness | G46SVL | PI | 0.030 | 0.018 |  | 1.730 | 0.107 | [-0.008, 0.068] |
| Length pair robustness | G46SVL | MS | -0.015 | 0.016 |  | -0.931 | 0.369 | [-0.049, 0.019] |
| Length pair robustness | G46SVL | Maternal BCI | 0.002 | 0.013 |  | 0.172 | 0.866 | [-0.027, 0.031] |
| Length pair robustness | G46SVL | R | -0.020 | 0.015 |  | -1.292 | 0.219 | [-0.053, 0.013] |
| Length pair robustness | G46TIL | PS | 0.005 | 0.016 |  | 0.305 | 0.765 | [-0.030, 0.039] |
| Length pair robustness | G46TIL | Paternal BCI | -0.006 | 0.014 |  | -0.454 | 0.657 | [-0.037, 0.024] |
| Length pair robustness | G46TIL | PI | 0.033 | 0.018 |  | 1.876 | 0.083 | [-0.005, 0.072] |
| Length pair robustness | G46TIL | MS | -0.006 | 0.016 |  | -0.393 | 0.700 | [-0.041, 0.028] |
| Length pair robustness | G46TIL | Maternal BCI | -0.014 | 0.014 |  | -1.013 | 0.329 | [-0.043, 0.016] |
| Length pair robustness | G46TIL | R | -0.033 | 0.016 |  | -2.098 | 0.056 | [-0.066, 0.001] |
| Width pair robustness | G25BW | PS | 0.027 | 0.025 |  | 1.109 | 0.288 | [-0.026, 0.081] |
| Width pair robustness | G25BW | Paternal BCI | -0.026 | 0.022 |  | -1.186 | 0.257 | [-0.074, 0.021] |
| Width pair robustness | G25BW | PI | 0.003 | 0.028 |  | 0.095 | 0.925 | [-0.057, 0.062] |
| Width pair robustness | G25BW | MS | -0.013 | 0.025 |  | -0.525 | 0.608 | [-0.066, 0.040] |
| Width pair robustness | G25BW | Maternal BCI | 0.004 | 0.021 |  | 0.193 | 0.850 | [-0.041, 0.049] |
| Width pair robustness | G25BW | R | 0.012 | 0.024 |  | 0.497 | 0.628 | [-0.040, 0.064] |
| Width pair robustness | G42BW | PS | 0.006 | 0.012 |  | 0.498 | 0.627 | [-0.019, 0.031] |
| Width pair robustness | G42BW | Paternal BCI | -0.005 | 0.010 |  | -0.457 | 0.655 | [-0.027, 0.018] |
| Width pair robustness | G42BW | PI | -0.012 | 0.013 |  | -0.895 | 0.387 | [-0.040, 0.016] |
| Width pair robustness | G42BW | MS | 0.007 | 0.012 |  | 0.615 | 0.549 | [-0.018, 0.032] |
| Width pair robustness | G42BW | Maternal BCI | -0.020 | 0.010 |  | -2.014 | 0.065 | [-0.041, 0.001] |
| Width pair robustness | G42BW | R | 0.019 | 0.011 |  | 1.651 | 0.123 | [-0.006, 0.043] |
| Width pair robustness | G46BW | PS | -0.009 | 0.016 |  | -0.602 | 0.558 | [-0.044, 0.025] |
| Width pair robustness | G46BW | Paternal BCI | -0.005 | 0.014 |  | -0.389 | 0.704 | [-0.036, 0.025] |
| **Width pair robustness** | **G46BW** | **PI** | **0.070** | **0.018** |  | **3.964** | **0.002** | **[0.032, 0.108]** |
| Width pair robustness | G46BW | MS | -0.014 | 0.016 |  | -0.859 | 0.406 | [-0.048, 0.021] |
| Width pair robustness | G46BW | Maternal BCI | -0.009 | 0.013 |  | -0.667 | 0.516 | [-0.038, 0.020] |
| **Width pair robustness** | **G46BW** | **R** | **-0.035** | **0.015** |  | **-2.258** | **0.042** | **[-0.068, -0.002]** |

Note: Sensitivity models replaced parental SVL and BCI with parental body mass, whereas pair-level robustness analyses were conducted at the clutch level. PM, paternal mass; MM, maternal mass; PI, paternal investment; R, parental relatedness coefficient; DD, developmental duration; V, growth rate; TIL, tibiofibula length; BW, body width. Confidence intervals are shown for fixed-effect estimates.

**Table S4**

Supplementary Table S4. Stage-interaction models testing whether associations between parental predictors and offspring traits differed among developmental stages. P values were adjusted across the exploratory interaction tests using the Benjamini-Hochberg false discovery rate (FDR) procedure.

| Trait group | Predictor | Interaction term | numDF | denDF | F | P value | FDR-adjusted q value |
| --- | --- | --- | --- | --- | --- | --- | --- |
| rate | PS | PS:Stage | 2 | 612 | 3.313 | **0.037** | 0.102 |
| **rate** | **Paternal BCI** | **Paternal BCI:Stage** | **2** | **612** | **14.283** | **8.66e-7** | **1.55e-5** |
| rate | PI | PI:Stage | 2 | 612 | 3.247 | **0.040** | 0.102 |
| rate | MS | MS:Stage | 2 | 612 | 4.466 | **0.012** | 0.053 |
| rate | Maternal BCI | Maternal BCI:Stage | 2 | 612 | 0.216 | 0.806 | 0.806 |
| rate | R | R:Stage | 2 | 612 | 2.156 | 0.117 | 0.191 |
| length | PS | PS:Stage | 3 | 822 | 0.407 | 0.748 | 0.801 |
| length | Paternal BCI | Paternal BCI:Stage | 3 | 822 | 2.087 | 0.100 | 0.181 |
| length | PI | PI:Stage | 3 | 822 | 0.901 | 0.440 | 0.528 |
| length | MS | MS:Stage | 3 | 822 | 2.247 | 0.081 | 0.163 |
| length | Maternal BCI | Maternal BCI:Stage | 3 | 822 | 2.519 | 0.057 | 0.128 |
| **length** | **R** | **R:Stage** | **3** | **822** | **5.979** | **4.94e-4** | **0.004** |
| width | PS | PS:Stage | 2 | 612 | 1.800 | 0.166 | 0.230 |
| width | Paternal BCI | Paternal BCI:Stage | 2 | 612 | 4.619 | **0.010** | 0.053 |
| width | PI | PI:Stage | 2 | 612 | 1.992 | 0.137 | 0.230 |
| width | MS | MS:Stage | 2 | 612 | 0.279 | 0.757 | 0.801 |
| width | Maternal BCI | Maternal BCI:Stage | 2 | 612 | 1.374 | 0.254 | 0.326 |
| width | R | R:Stage | 2 | 612 | 3.625 | **0.027** | 0.098 |

Note: Stage-interaction models tested whether the associations between parental predictors and offspring traits differed among developmental stages. PS, paternal SVL; MS, maternal SVL; BCI, body condition index; PI, paternal investment; R, parental relatedness coefficient.

**Table S5**

Supplementary Table S5. Variance inflation factors (VIFs) for the final models.

| Model folder | Response with max VIF | Term with max VIF | Max VIF |
| --- | --- | --- | --- |
| duration/model results duration main SVL BCI | G0-25DD | PI | 2.22 |
| duration/model results duration sensitivity mass | G0-25DD | PI | 2.16 |
| rate/model results rate main SVL BCI | G25-42V | PI | 2.93 |
| rate/model results rate sensitivity mass | G25-42V | PI | 2.67 |
| rate/model results rate pair level robustness | G0-25V | PI | 2.22 |
| body/model results body length main SVL BCI | G46TIL | PI | 3.61 |
| body/model results body length sensitivity mass | G46TIL | PI | 3.02 |
| body/model results body length pair level robustness | G25TL | PI | 2.22 |
| body/model results body width main SVL BCI | G46BW | PI | 3.57 |
| body/model results body width sensitivity mass | G46BW | PI | 3.02 |
| body/model results body width pair level robustness | G25BW | PI | 2.22 |

Note: VIF, variance inflation factor. The term with the highest VIF is reported for each model set. All VIF values were < 4, indicating no serious multicollinearity among predictors in the final models.
