# Supplementary material for: Parental traits, reproductive investment, and genetic relatedness shape offspring development and morphology in an explosive-breeding toad
Source: Front Zool. 2026 Aug 11;23:39. doi: 10.1186/s12983-026-00627-0 (PMC13750053; doi:10.1186/s12983-026-00627-0)
Supplement: Supplementary file 2 [file 12983_2026_627_MOESM2_ESM.docx]

**Supplementary Information**

**Additional file 2**

**Supplementary Figure S1**

Additional nominal stage-dependent associations between parental predictors and offspring traits in *Bufo gargarizans* that are not shown in Figure 3. (A) Association between maternal snout-vent length (SVL) and offspring growth rate across developmental stages. (B) Association between paternal investment and offspring growth rate across developmental stages. (C) Association between paternal body condition index (BCI) and offspring body width across developmental stages. (D) Association between parental relatedness and offspring body width across developmental stages. Lines show stage-specific fitted values from single-predictor stage-interaction models, shaded bands indicate 95% confidence intervals, and points show observed values.

**Supplementary Figure S2**

Pair-level robustness plot for the association between paternal investment and offspring body width at G46 in *Bufo gargarizans*. The solid line shows fitted values from the pair-level linear model, the shaded band indicates the 95% confidence interval, and points show pair-level partial residuals.
